# Effectiveness of Hypnobirthing, Music therapy and Combined Intervention in Reducing Low Back Pain Among Third-Trimester Pregnant Women: A Quasi-Experimental Study

**DOI:** 10.12688/f1000research.168854.1

**Published:** 2025-09-03

**Authors:** Murbiah, Santhna Letchmi Panduragan, Kasmahwati Pardi, Hafizah Che hassan, Husin

**Affiliations:** 1 Lincoln university Collage, Lincoln University College Faculty of Medicine, Petaling Jaya, Selangor, Malaysia; 2 universitas muhammdiyah ahamd dahlan, universitas muhammadiyah ahmad dahlan palembang, palembang, sumatera selatan, Indonesia; 3 Sekolah tinggi ilmu kesehatan Bina Husada, sekolah tinggi llmu kesehatan Bina Husada, Palembang, Sumatera selatan, Indonesia

**Keywords:** Pregnancy, Low back pain, Hypnobirthing, Music therapy, Third trimester

## Abstract

**Background:**

Low back pain (LBP) is a common complaint experienced by pregnant women, especially in the third trimester, which can interfere with daily activities, sleep quality, and readiness for labor. Nonpharmacological approaches such as music therapy and hypnobirthing are developing as alternatives that are considered safe, inexpensive, and minimal in side effects, but the effectiveness of the combination of the two methods has not been widely studied in the context of pregnancy in Indonesia

**Methods:**

This quasi-experimental study aimed to examine the effectiveness of hypnobirthing, music therapy, and their combination in alleviating LBP among third-trimester pregnant women. A total of 200 participants were divided into four groups: hypnobirthing (n=50), music therapy (n=50), a combination group (n=50), and a control group receiving standard antenatal care (n=50). Pain levels were assessed before and after the intervention using the Visual Analog Scale (VAS).

**Results:**

The study showed a statistically significant reduction in LBP intensity in all the intervention groups compared to that in the control group (p < 0.001), with the combined intervention group demonstrating the greatest improvement. These findings suggest that both hypnobirthing and music therapy are effective in managing pregnancy-related LBP, and their combination may offer enhanced benefits for managing pregnancy-related LBP.

**Conclusions:**

This study supports the inclusion of non-pharmacological approaches in routine antenatal care to improve maternal comfort and well-being.

## Introduction

Low back pain during pregnancy is one of the most common musculoskeletal complaints experienced by women worldwide, particularly during the third trimester. Based on a global meta-analysis of 28 studies with a total of 12,908 respondents, the prevalence of lower back pain during pregnancy was 40.5%, with a peak of 47.8% in the third trimester ( Omoke et al., 2021). This condition not only causes physical discomfort but also reduces the quality of life of pregnant women, limits their daily activities, and increases the risk of medical intervention during labor. Amidst the development of non-pharmacological approaches in nursing practice, music therapy and hypnobirthing are gaining attention as safe, effective, and evidence-based alternative interventions. However, there is limited integration of these approaches in maternal health care policies globally, which calls for rigorous ( Purnamasari, 2021).

Pregnant women experience discomfort during the adaptation process due to physical and psychological changes. Physiological changes in pregnant women throughout pregnancy can have harmful effects. Low back pain (LBP) during pregnancy is a significant global health issue, ranking as the highest cause of disability worldwide over the past three decades. According to the 2021 Global Burden of Disease study, over 628 million people worldwide experience LBP, with the highest prevalence occurring in women of reproductive age, especially during pregnancy (Li et al., 2024). Although the global prevalence rate has decreased in relative terms, the absolute number of sufferers has increased significantly because of population growth and increased life expectancy. The World Health Organization (WHO) and several international studies have shown that more than 50% of pregnant women experience LBP, especially in the third trimester, which interferes with daily activities and potentially worsens psychological conditions during pregnancy ( Chen et al., 2021).

Back pain during pregnancy can be caused by weight gain, changing posture, hormone fluctuations, shifting the center of gravity, muscle separation, the natural elasticity of ligaments to prepare for labor, and stress. Lower back discomfort in pregnant women is frequently caused by mechanical instability of the lumbar spine and pelvis. The lumbar joints, ligaments, and muscles are overstressed as a result of compensatory lumber lordosis ( Atis & Rathfisch, 2018). Pregnancy hormone changes cause the ligaments connecting the pelvic bones to the spine to relax and the joints to become less rigid. This results in pain when bending, lifting, walking, standing, and sitting for extended periods of time ( Amayri et al., 2023).

This phenomenon not only limits the physical activities of pregnant women but also affects their quality of life and psychological state to a large extent. There is an increasing need for safe, sustainable, and non-pharmacological approaches to pain management ( Manyozo et al., 2019). This condition impacts individuals and adds to the burden of the global health system, given the importance of supporting healthy pregnancies as an essential component of human development. In the academic realm, various non-pharmacological intervention approaches, including music therapy and hypnobirthing, have been developed as part of the integration of health technology, culture-based interventions, and psychological approaches to pain mitigation during pregnancy. This development calls for more profound research to empirically and contextually assess the effectiveness of these approaches ( Salamah, 2019).

National data from the Indonesian Ministry of Health and other regional studies indicate a substantial prevalence of lower back pain (LBP) among pregnant women in Indonesia, particularly the third trimester. A regional survey indicated that over 60% of pregnant women in South Sumatra experienced back discomfort that impacted their daily activities and sleep quality ( Ismawati, 2022). Research in several government hospitals shows that most pain complaints are not managed through evidence-based intervention methods, and pharmacological interventions are often avoided due to risks to the fetus. Despite policies on maternal and child health, interventions for low back pain during pregnancy have not been prioritized in the primary healthcare system. This gap highlights the need for in-depth studies on alternative therapies that are highly effective, safe, and widely applicable. In addition, community-based approaches and health system support need to be designed based on robust and contextualized research results, making this research important not only for theory development but also for the reformulation of maternal care policies at the national level ( Bloom & Reenen, 2021).

In the third trimester, nursing care for pregnant women with back pain complaints and acute pain problems can be provided through non-pharmacological pain management interventions with distraction and diversion through classical music therapy and hypnobirthing relaxation techniques. Non-pharmacological pain management methods, such as deep breathing exercises, are frequently employed in hospitals ( Hu et al., 2020). Typically, doctors use medications to relieve pain. Although much research has been conducted on the influence of music on physical and emotional well-being, there is still a lack of research specifically exploring the effects of traditional Indonesian music. Hypnobirthing and music therapy have emerged as promising complementary therapies for managing pain and anxiety during pregnancy and labor. However, empirical evidence comparing their effectiveness, both individually and in combination, in reducing LBP during late pregnancy remains limited (Thakare et al., 2022).

The urgency of this research is highlighted by two main aspects: its contribution to the development of maternity nursing science and its potential implementation in primary health care systems. From an academic perspective, this study enables the renewal of intervention models in nursing based on cultural integration and relaxation psychology. From a practical point of view, the findings of this study can help create standard procedures for music therapy and hypnobirthing that can be used in health centers, especially in cities such as Palembang ( Sr et al., 2021). In addition, this study contributes to national efforts to reduce the use of pharmacological interventions during pregnancy, which, in the long run, impacts maternal and fetal safety and health system efficiency. This study aimed to evaluate the effectiveness of hypnobirthing, music therapy, and their combination in reducing lower back pain among third-trimester pregnant women.

## Methods

### Study area

This study will be conducted in four primary health care centers (PUSKESMAS) spread across the administrative area of Palembang City, South Sumatra Province, Indonesia. Palembang City was chosen as the study location because it has a large population of pregnant women and socio-cultural variations that reflect the diversity of urban community contexts in Indonesia. The selection of Puskesmas as the main research site was based on the role of this institution as a first-level healthcare facility that serves as the starting point for pregnancy and childbirth services for the community. In addition, the presence of midwives and health workers at Puskesmas allows the implementation of therapeutic music and hypnobirthing interventions in a standardized manner, in accordance with applicable medical procedures (Sari et al., 2023).

The study is scheduled to last for five months, from October 2024 to February 2025 and consists of the following stages: October 2024 Instrument development and validation, November 2024: submission and approval of ethical clearance by the institutional ethics committee, and acquisition of official research permits from the Indonesian Department of Health and PUSKESMAS. December 2024: Participant recruitment and eligibility screening based on predetermined inclusion and exclusion criteria. January 2025 Intervention phase, involving the administration of music therapy and hypnobirthing sessions for four weeks under the supervision of certified health personnel. February 2025 post-intervention data collection, statistical data processing, and preliminary interpretation of findings.

### Study design

A quasi-experimental design with randomized group allocation was used to assess the effectiveness of non-pharmacological interventions for reducing lower back pain in third-trimester pregnant women. This study involved four parallel groups that received different interventions (Polit & Beck, 2014; Yulius, 2023).

### Inclusion and exclusion criteria of participants

The inclusion criteria were as follows: third-trimester pregnant women, experiencing moderate to severe lower back pain, residing in Palembang City, Willing to participate in the full intervention and evaluation process. Exclusion criteria: Experiencing pregnancy complications requiring special care unwilling to participate in the full intervention, having hearing or psychiatric disorders that could influence intervention outcomes

### Recruitment

Sampling is based on the selection of random units from a population. Sampling was carried out using a simple random sampling method, which emphasizes that each member of the population has the same chance/probability of being selected as a research sample ( Yulius, 2023). The sample selection process is carried out through initial screening using a low back pain identification questionnaire based on the Visual analog scale (VAS). Respondents who meet the threshold of moderate to severe pain will be given an explanation of the purpose of the study and intervention procedures, then asked to sign an informed consent. A total of 200 third-trimester pregnant women were recruited from the participating health centers using random selection. Participants were then randomly assigned to four groups (n = 50 per group): hypnobirthing in music therapy intervention group, the combined hypnobirthing and music therapy group, and the control group, which received routine antenatal care only leaflet. The sample was determined using a random technique (probability sampling method). 

### Sample size

The sample size in this study was determined and calculated using G*Power ( Erdfelder, 2007) based on Cohen’s formula ( Brydges, 2019) at a significance level of 0.05 and a desired power of 0.90. The estimated effect size was 0,80, derived from a previous study using one-way analysis of variance (ANOVA). The sample size for each group was 41, with a 20% dropout rate, resulting in a total sample size of 50 for each group. Thus, the total number of participants was approximately 200. The sample was proportionally divided into four groups: a music therapy group, a hypnobirthing group, a combination group of hypnobirthing intervention with music therapy, and a control group. The choice of this technique strengthens the statistical power of the data analysis and ensures that the sample size is large enough to detect statistically significant differences between groups.

### Outcome

The primary outcome of the study was the reduction in lower back pain intensity before and after a four-week intervention period. Pain intensity was measured using a Visual Analog Scale (VAS).

### Tools for collecting information

This study used various instruments to measure and intervene in low back pain in pregnant women in their third trimester. Each instrument was designed to ensure the validity and effectiveness of the results in accordance with the quasi-experimental pre- and post-test approach. The instruments used were as follows:

### Questionnaire

The VAS questionnaire was used to measure the level of lower back pain experienced by third-trimester pregnant women before and after the intervention. The VAS was chosen because it is one of the most widely used pain measurement instruments in clinical research and maternity nursing. This scale allows respondents to provide a subjective assessment of pain intensity on a numerical scale from 0 to 10, where 0 indicates no pain and 10 indicates severe pain. The instrument has been shown to have high construct validity in various clinical and nursing contexts and is compatible with quasi-experimental designs that require a systematic comparison of pre- and post-intervention scores ( Bryndal et al., 2020). The VAS has been shown to have high construct validity and reliability in a variety of clinical contexts, including maternity nursing (Andreyani & Bhakti, 2023).

### Hypnobirthing intervention

Hypnobirthing intervention is carried out in accordance with standard procedures included guided breathing, visualization, and deep relaxation techniques conducted by a certified practitioner twice weekly for four weeks (Saad & Jariyah, 2022).

### The music therapy intervention

The music therapy intervention was conducted according to the standard procedure of 30 min of daily listening to relaxing instrumental music for 4 weeks. (Saad & Jariyah, 2022), the music used is the traditional “Gending Sriwijaya” composition. This piece, created by Nungcik AR in 1943 and accompanied by Palembang-style gamelan music arranged by A. Dahlan Rachim and R.C. Hardjosubroto is a cultural symbol of South Sumatra. The traditional music of Gending Sriwijaya used in this research is part of the traditional cultural heritage of the people of Palembang, South Sumatra, Indonesia, which is protected by Law Number 28 of 2014 concerning Copyright, specifically related to traditional cultural expressions. the instrumental version of Gending Sriwijaya was published by Sangsaka sound system on June 23, 2019 through the youtube https://www.youtube.com/watch?v=V8lp3F0LoS0&list=RDV8lp3F0LoS0&start_radio=1. The version does not violate any party’s copyright or license, and its use is intended purely for non-commercial research purposes. The use of Gending Sriwijaya in this research aims to be a form of music therapy intervention that promotes and respects local culture as part of a local wisdom-based approach in maternal health services. The music will not be used outside of academic and scientific interests, will not be used for commercial purposes, and will not change the lyrics or original meaning of the song in a way that could degrade cultural values. Combination group: received a joint intervention combining hypnobirthing techniques and therapeutic music under the same schedule and procedures. Zenodo. Questionnaire and research tools. https://doi.org/10.5281/zenodo.16881327 ( Murbiah, 2025a).

### The leaflet

The leaflet was given once at the beginning of the intervention (week 1) and briefly discussed by the staff as a form of educational control to control group. Zenodo. Questionnaire and research tools. https://doi.org/10.5281/zenodo.16881327 ( Murbiah, 2025a).

### Statistical analysis

This study employed a quantitative approach that utilized a pre-test and post-test quasi-experimental design, which included a control group. Therefore, the data analysis techniques used included descriptive and inferential statistical analyses. Descriptive statistics were used to describe the characteristics of the respondents, such as maternal age in years, gestational age in weeks, education, occupation, parity, and pre- and post-intervention pain scores. The data are presented as mean, standard deviation, minimum, maximum, and frequency distribution. Inferential statistics were used to test the effectiveness of the intervention, using a paired sample t-test to compare pain scores before and after the intervention in the same group and an independent sample t-test or ANOVA to determine differences between treatment groups, namely music therapy, hypnobirthing, and a combination of both ( Yulius, 2023).

## Results

The research phase began with the approval of the ethics committee and research permit in July 2024. Researchers began to identify respondents based on the following inclusion criteria: pregnant women in the third trimester, experiencing mild to severe lower back pain, and primigravida. After the respondents were collected, lower back pain was measured using a visual analogue scale (VAS) questionnaire on 200 respondents of pregnant women in the third trimester in the working areas of the Pembina Health Center, Nagaswidak Health center, Taman Bacaan Health Center and Pembina Health Center. Data analysis was performed as follows:

Based on Table 1, a total of 200 respondents were involved in this study. The majority of respondents were in the age range of 21-25 years (56%), indicating that most mothers were at the optimal reproductive age. The highest gestational age was in the range of 28-31 weeks (70.5%), indicating that the respondents were in the third trimester phase, which generally experiences an increase in the number of pregnancies.

**Table 1.** Sociodemographic characteristics of participants at baseline.

Baseline characteristics	Music therapy group		Hypnobirthing group		Combination group		Control group		Full sample	
	N	%	N	%	N	%/	N	%	N	%
Age (years)										
15-20	1	0.5	0	0	3	1.5	1	0.5	5	2.5
21-25	30	15	31	15.5	28	14	23	11.5	112	56
26-30	13	6.5	16	8	10	5	16	8	55	27.5
31-35	6	3	3	1.5	9	4.5	9	4.5	27	13.5
36-40	0	0	0	0	0	0	0	0	0	0
41-45	0	0	0	0	0	0	1	0.5	1	0.5
Pregnancy Age (Weeks)										
24-27	1	0.5	0	0	0	0	0	0	1	0.5
28-31	36	18	35	17.5	35	17.5	35	17.5	141	70.5
32-35	13	6.5	15	7.5	15	7.5	15	7.5	58	29
Education										
Middle School	7	14	10	20	10	20	12	24	39	19.5
High School	33	66	32	64	33	66	30	60	128	64
University	10	20	8	16	7	14	8	16	33	16.6
Work										
Wife House	41	82	42	84	40	80	38	76	161	80.5
Working	9	18	8	16	10	20	12	24	39	19.5
Marital Status										
Single	0	0	0	0	0	0	0	0	0	0
Married	50	100	50	100	50	100	50	100	200	100

Based on Table 2, the average pain level before music therapy was 5,96 (SD = 1,958), and the average pain level after music therapy was 4,48 (SD =1,644). The average pain level before Hypnobirthing was 6,50 (SD = 1,619, the average pain level after music therapy was 4,50 (SD =1,619), and the average pain level before Hypnobirthing with music therapy was 5,50 (SD = 1,669. The average pain level after music therapy was 3,52 (SD =1,644), and the average pain level in the control group was 5,22 (SD = 1,075. The average pain level after control group was 4,56 (SD =1,280).

**Table 2.** Distribution of low back pain score pre and post intervention.

Intervention	N	Pre intervention			Post intervention		
		Mean	SD	Min-Max	Mean	SD	Min-Max
Hypnoburthing group	50	5.96	1,958	3-9	4.48	1,644	2-7
Music therapy group	50	6.50	1,619	4-9	4.50	1,619	2-7
Combination group	50	5.50	1,669	3-8	3.52	1,644	1-6
Control group	50	5.22	1,075	4-7	4.56	1,280	2-7

Based on Table 3, the average pain score before the combined therapy (hypnobirthing and music therapy) was 5,50 (SD = 1,669), indicating moderate to severe pain levels. After the combined intervention, the average pain score decreased to 3,52 (SD = 1,644), showing a significant reduction to mild pain levels. The mean difference in pain scores (pre-vs. post-test) was 1,803 (SD = 2,157), demonstrating a substantial reduction in pain following the combined therapy. The paired t-test statistic (t(49) = 22,496) was highly significant (p 0.000). This confirms that the observed reduction in pain scores was not due to chance but was likely caused by the combined effect of hypnobirthing and music therapy.

**Table 3.** Paired Sample t-Test for low back pain score before and after intervention (per Group).

Variable	N	Low back pain (Pre)		Low back pain (Post)		T	df	P value	Cohen’s d
		M	SD	M	SD				
Music Therapy	50	5.96	1,958	4.48	1,644	10,984	49	0.000	1.553
Hypnobirthing	50	6.50	1,619	4.50	1,619	14,289	49	0.000	2.503
Combination	50	5.50	1,669	3.52	1,644	22,496	49	0.000	3.865
Control	50	5.22	1,075	4.56	1,280	5,505	49	0.000	1.092

Based on Table 4, the ANOVA test revealed a statistically significant difference in the effectiveness of the interventions (F = 20.521, p: 0,000) This indicating that at least one group differed significantly in terms of pain reduction.

**Table 4.** One Way ANOVA summary for pain reduction between intervention group and control group.

Variable	SS	Df	Mean Square	F	P
Between groups	73.600	3	24.533	20.521	0.000
Within groups	234.320	196	1.196		
Total	307.929	199			

Based on Table 5, the average pain score before the combined therapy (hypnobirthing and music therapy) was 5,50 (SD = 1,669), indicating moderate to severe pain levels. After the combined intervention, the average pain score decreased to 3,52 (SD = 1,644), showing a significant reduction to mild pain levels. The mean difference in pain scores (pre-test vs. post-test) was 1,803 (SD = 2,157), demonstrating a substantial reduction in pain following the combined therapy. The paired t-test statistic (t(49) = 22,496) was highly significant with p: 0.000. This confirms that the observed reduction in pain scores was not due to chance but was likely caused by the combined effect of hypnobirthing and music therapy.

**Table 5.** Post hoc comparisons using Tukey HSD test for mean pain scores between the groups.

Group 1	Group 2	Mean Difference	95%CI	P	Significant
Music Therapy	Hypnobirthing	-0.080	(-0.65 – 0.49)	0.980	No
Music Therapy	Combination	0.160	(-0.41 – 0.73)	0.884	No
Music Therapy	Control	-1.360	(-1.93 – -0.79)	0.000	Yes
Hypnobirthing	Music Therapy	0.080	(-0.49 – 0.65)	0.983	No
Hypnobirthing	Combination	0.240	(-0.33 – 0.81)	0.691	No
Hypnobirthing	Control	-1.280	(-1.85 – -0.71)	0.000	Yes
Combination	Music Therapy	-0.160	(-0.73 – 0.41)	0.884	No
Combination	Hypnobirthing	-0.240	(-0.81 – 0.33)	0.691	No
Combination	Control	-1.520	(-2.09 – -0.95)	0.000	Yes
Control	Music Therapy	1.360	(0.79 – 1.93)	0.000	Yes
Control	Hypnobirthing	1.280	(0.71 – 1.85)	0.000	Yes
Control	Combination	1.520	(0.95 – 2.09)	0.000	Yes

## Discussion

The findings demonstrate that both hypnobirthing and music therapy are effective in reducing LBP during the third trimester of pregnancy. The superior effectiveness of the combined intervention suggests potential synergistic benefits when these approaches are used concurrently. These results align with previous studies, highlighting the efficacy of relaxation-based therapies in managing prenatal discomfort. Integrating these non-invasive, low-cost interventions into antenatal care protocols may enhance maternal comfort, reduce dependence on pharmacological pain relief, and support holistic prenatal care. Saad and Jariyah (2022) found that hypnobirthing works through the cognitive-emotional pathway by reducing anxiety and activating positive suggestion mechanisms. Therefore, when these two approaches are combined, the result is not simply a summation of effects but a synergy that strengthens the body’s adaptive response to pain. This aligns with the biopsychosocial approach to pain management, which emphasizes the importance of integrating biological, psychological, and sociocultural interventions ( Maidawilis et al., 2023).

Comparison between the three intervention groups showed that the combination of hypnobirthing and music therapy provided the best results in reducing lower back pain, followed by single hypnobirthing and single music therapy. This finding indicates that integrating non-pharmacological methods with different but complementary approaches can strengthen the effect of interventions on pain. This combination of methods addresses pain perception from two directions: cognitive-affective through hypnobirthing and sensory-emotional through music ( Asmara et al., 2017).

In terms of implementation, these results show that combined interventions have enormous potential for application in midwifery practice in Indonesia, especially in non-invasive antenatal care. The application of a multimodal approach that combines cultural and psychological aspects provides a more holistic therapeutic response and empowers patients. This effectiveness is the basis for developing an integrated intervention model in the management of musculoskeletal pain in pregnancy ( Hall et al., 2016).

Nursing care for pregnant women with back pain and acute pain issues may include non-pharmacological pain management therapies, such as distraction and diversion through classical music therapy and hypnobirthing relaxation techniques. Non-pharmacological pain management techniques, such as deep breathing exercises, are commonly used in hospitals. Medications are generally employed exclusively for pain relief ( Cheung et al., 2023). Despite extensive research on the impact of music on physical and emotional health, there remains a deficiency of studies explicitly examining the effects of traditional Indonesian music, such as Gending Sriwijaya, on lower back pain in pregnant women ( Santiváñez-Acosta et al., 2020).

This synergy amplifies the release of endorphins and oxytocin, inhibits cortisol production, and decreases the perception of the threat often associated with pregnancy pain. These combined effects create optimal physical and psychological conditions for a sustained reduction in pain. In other words, the combination of interventions provides a layered stimulus that supports the mother’s self-regulation capacity for pain ( Sr et al., 2021).

Furthermore, the combined intervention of hypnobirthing and therapeutic music works through physiological pathways and creates profound psychospiritual effects on pregnant women. In hypnobirthing, pregnant women are directed to enter a deeper state of relaxed awareness through positive affirmation and visualization techniques ( Sr et al., 2021). This process maximizes the emotional and cognitive resonance effects when paired with traditional music, such as Gending Sriwijaya. Traditional music has a stable and harmonious rhythmic and melodic structure, which is known to facilitate light trance states that are ideal for the reception of positive suggestions during hypnobirthing sessions.

## Conclusion

Hypnobirthing and music therapy significantly reduced pregnancy-related lower back pain, with their combination yielding the most pronounced benefits. These non-pharmacological interventions should be considered for routine use in third-trimester prenatal care to improve maternal wellbeing. All interventions were effective, and a combined approach may offer better benefits or be a promising holistic approach.

## Ethical considerations

This study was approved by head of ethics review committee Dr. Suzanna, S.Kep., Ns., M. Kep on 25 November 2024 by Health Research Ethics Committee of IKesT Muhammadiyah Palembang have granted ethical approval No: 002009/KEP IKesT Muhammadiyah Palembang/2024). The approval is based on 7 (seven) WHO 2011 Standard and guidance part III ( WHO, 2011), Namely Ethical basis for decision-making will reference to the fulfilment of 2016 CIOMS guideline ( CIOMS, 2016). The respondents were third-trimester pregnant women who experienced mild to severe lower back pain. Prior to participation, all eligible pregnant women were informed about the study objectives, procedures, potential risks, and benefits. Written informed consent was obtained from all participants before data collection began. The consent process emphasized voluntary participation, the right to withdraw at any stage without any consequences, and assurance of confidentiality. No parental or guardian consent was required since all participants were adults above the age of 18 years. All collected data were anonymized to ensure participant privacy and confidentiality, and after being explained, the respondents are asked to sign the availability of being a respondent on the consent sheet provided. Zenodo. Questionnaire and research tools. https://doi.org/10.5281/zenodo.16881327 ( Murbiah, 2025a).

## Data Availability

Zenodo. Research data (DataSet). https://doi.org/10.5281/zenodo.16758814 ( Murbiah, 2025b). Data are available under the terms of the Creative Commons Attribution 4.0 International license (CC-BY 4.0). This Project contains the following underlying data: Research Dataset (DataSet) This file includes raw and cleaned numerical data on low back pain scores (VAS), demographic characteristics, and intervention groups (music therapy, hypnobirthing, combined intervention, and control) from 200 third-trimester pregnant women participants. Data are available under the terms of the Creative Commons Attribution 4.0 International license (CC-BY 4.0).

## References

[ref1] Amayri A Khalayli N Haj Ali D : Low back pain in a sample of Syrian pregnant women: A cross-sectional study. *Health Sci. Rep.* 2023;6(7):e1389. 10.1002/hsr2.1389 37408868 PMC10318381

[ref2] Asmara MS Rahayu HE Wijayanti K : Efektifitas Hipnoterapi dan Terapi Musik Klasik Terhadap Kecemasan Ibu Hamil Resiko Tinggi di Puskesmas Magelang Selatan Tahun 2017. *Urecol.* 2017;329–334. Reference Source

[ref3] Atis FY Rathfisch G : The effect of hypnobirthing training given in the antenatal period on birth pain and fear. *Complement. Ther. Clin. Pract.* 2018;33(July):77–84. 10.1016/j.ctcp.2018.08.004 30396631

[ref4] Bloom N Reenen J Van : Manajemen Nyeri Persalinan Non Farmakologis. *NBER Working Papers.* 2021. Reference Source

[ref5] Brydges CR : Effect Size Guidelines, Sample Size Calculations, and Statistical Power in Gerontology. *Innov. Aging.* 2019;3(4):1–8. 10.1093/geroni/igz036 PMC6736231 31528719

[ref6] Bryndal A Majchrzycki M Grochulska A : Risk factors associated with low back pain among a group of 1510 pregnant women. *J. Pers. Med.* 2020;10(2):1–10. 10.3390/jpm10020051 32549306 PMC7354496

[ref7] Chen L Ferreira ML Beckenkamp PR : Comparative Efficacy and Safety of Conservative Care for Pregnancy-Related Low Back Pain: A Systematic Review and Network Meta-analysis. 2021;1–13. 10.1093/ptj/pzaa200 33210717

[ref8] Cheung PS McCaffrey T Tighe SM : Music as a health resource in pregnancy: A cross-sectional survey study of women and partners in Ireland. *Midwifery.* 2023;126(June):103811. 10.1016/j.midw.2023.103811 37708586

[ref9] CIOMS: The Council for International Organizations of Medical Sciences (CIOMS) announces the publication of International ethical guidelines for health-related research involving humans. 2016. 10.56759/rgxl7405 40523065

[ref10] Erdfelder E : G * Power 3: A flexible statistical power analysis program for the social, behavioral, and biomedical sciences. 2007;39(2):175–191. 10.3758/bf03193146 17695343

[ref11] Hall H Cramer H Sundberg T : The effectiveness of complementary manual therapies for pregnancy-related back and pelvic pain A systematic review with meta-analysis. *Medicine (United States).* 2016;95(38):e4723. 10.1097/MD.0000000000004723 27661020 PMC5044890

[ref12] Hu X Ma M Zhao X : Effects of exercise therapy for pregnancy-related low back pain and pelvic pain: A protocol for systematic review and meta-analysis. *Medicine (United States).* Lippincott Williams and Wilkins;2020;99(3). 10.1097/MD.0000000000017318 PMC7220333 32011431

[ref13] Ismawati S : Pijat Hamil dan Relaksasi Nafas Dalam. 2022;1:48–56. 10.33860/njb.v1i1.1049

[ref22] Koukoulithras I Sr Stamouli A Kolokotsios S : The Effectiveness of Non-Pharmaceutical Interventions Upon Pregnancy-Related Low Back Pain: A Systematic Review and Meta-Analysis. *Cureus.* 2021;13(1):e13011–e13011. 10.7759/cureus.13011 33728108 PMC7934802

[ref14] Maidawilis Asmaria M Alimuddin : Reducing back pain intensity using a combination of music therapy and yoga: Case study in pregnant women. *J. Phys. Educ. Sport.* 2023;23(12):3385–3390. 10.7752/jpes.2023.12388

[ref15] Manyozo SD Nesto T Bonongwe P : Low back pain during pregnancy: Prevalence, risk factors and association with daily activities among pregnant women in urban Blantyre, Malawi. *Malawi Med. J.* 2019;31(1):71–76. 10.4314/mmj.v31i1.12 31143400 PMC6526334

[ref16] Murbiah: Questionnaire and research tools. 2025a. 10.5281/zenodo.16881327

[ref17] Murbiah: Research data.(DataSet).2025b. 10.5281/zenodo.16758814

[ref18] Omoke NI Amaraegbulam PI Umeora OUJ : Prevalence and risk factors for low back pain during pregnancy among women in Abakaliki, Nigeria. *Pan Afr. Med. J.* 2021;39:70. 10.11604/pamj.2021.39.70.24367 34422193 PMC8363951

[ref19] Purnamasari KD : Gambaran Tingkat Nyeri Punggung Pada Ibu Hamil Trimester 2 Dan 3 Di Wilayah Kerja Puskesmas Buleleng 1 Tahun 2021. *J. Midwifery Public Health.* 2021;1(1):9. 10.25157/jmph.v1i1.2000

[ref20] Salamah U : Hubungan Pengetahuan dan Sikap Remaja Putri terhadap Perilaku Penanganan Dismenore. *Jurnal Ilmiah Kebidanan Indonesia.* 2019;9(03):123–127. 10.33221/jiki.v9i03.382

[ref21] Santiváñez-Acosta R Tapia-López E d l N Santero M : Music therapy in pain and anxiety management during labor: A systematic review and meta-analysis. *Medicina (Lithuania).* 2020;56(10):1–11. 10.3390/medicina56100526 33050409 PMC7599829

[ref23] WHO: *Standards and operational guidance for ethics review of health-related research with human participants.* Geneva: World Health Organization;2011. Reference Source 26269877

[ref24] Yulius T : *Metodologi Penelitian: Langkah Praktis (Pemula) Merancang Penelitian Kuantitatif.* Trans Info Media;2023.

