# Supplementary material for: Effectiveness of Hypnobirthing, Music therapy and Combined Intervention in Reducing Low Back Pain Among Third-Trimester Pregnant Women: A Quasi-Experimental Study
Source: F1000Res. 2026 Jan 13;14:868. Originally published 2025 Sep 3. [Version 2] doi: 10.12688/f1000research.168854.2 (PMC12584645; doi:10.12688/f1000research.168854.2)
Supplement: Peer review history

# Reviewer report: Reviewer response for version 2

**DOI:** 10.5256/f1000research.194657.r450032

**Published:** 2026-02-06

**Authors:** Bayu Anggileo Pramesona

**Affiliations:** 1 Department of Public Health, Faculty of Medicine, Lampung University (Universitas Lampung), Bandar Lampung, Indonesia

Thank you for your great effort to revise the manuscript. I think all my comments have been addressed properly by the authors, and I have no further comments. Thank you.

Is the work clearly and accurately presented and does it cite the current literature?

Yes

If applicable, is the statistical analysis and its interpretation appropriate?

Partly

Are all the source data underlying the results available to ensure full reproducibility?

Yes

Is the study design appropriate and is the work technically sound?

Yes

Are the conclusions drawn adequately supported by the results?

Yes

Are sufficient details of methods and analysis provided to allow replication by others?

Partly

Reviewer Expertise:

Health policy, public health nursing, healthcare management

I confirm that I have read this submission and believe that I have an appropriate level of expertise to confirm that it is of an acceptable scientific standard.

# Reviewer report: Reviewer response for version 2

**DOI:** 10.5256/f1000research.194657.r450031

**Published:** 2026-01-23

**Authors:** Abu R. Srinivasan

**Affiliations:** 1 Sri Balaji Vidyapeeth (Deemed to be University), Pillayarkuppam, Puducherry, India

The authors had taken due cognizance of the comments/suggestions/pertinent points made by this Reviewer and have since amended the initial version of the manuscript suitably and appropriately, to the best possible and optimal extent, in keeping with the objectivity and reliability of the study. Furthermore, the manuscript in the present form appears quite robust which would also pave way for further studies in research, related to evidence based medicine.

 The study affords immense potentials in the promulgation of integrated and tailored interventions that would go a long way.

 In addition, studies related to comparisons, in future, utilizing different genres of music would generate novel findings. Also, the present study has opened newer vistas for carrying out  further comprehensive studies using mixed methods and qualitative research centering around fetal and neonatal outcomes.

Is the work clearly and accurately presented and does it cite the current literature?

Partly

If applicable, is the statistical analysis and its interpretation appropriate?

Partly

Are all the source data underlying the results available to ensure full reproducibility?

Yes

Is the study design appropriate and is the work technically sound?

Partly

Are the conclusions drawn adequately supported by the results?

Partly

Are sufficient details of methods and analysis provided to allow replication by others?

Partly

Reviewer Expertise:

Biochemistry related to metabolism and metabolic disorders, Endocrine Biochemistry, Music as an adjuvant therapeutic modality, Diagnostic enzymology

I confirm that I have read this submission and believe that I have an appropriate level of expertise to confirm that it is of an acceptable scientific standard.

# Reviewer report: Reviewer response for version 1

**DOI:** 10.5256/f1000research.186071.r412885

**Published:** 2025-10-23

**Authors:** Bayu Anggileo Pramesona

**Affiliations:** 1 Department of Public Health, Faculty of Medicine, Lampung University (Universitas Lampung), Bandar Lampung, Indonesia

Abstract:

 Methods:

1. Where was the study setting and when the study was conducted?
2. What the statistical analysis used in this study?

Conclusion:

 It should be more specific and refers to your study objective.

 Introduction.

1. What is the urgency to do this study in the primary health care setting?

Methods:

1. “This study will be conducted in four primary health care centers (PUSKESMAS) spread across the administrative area of Palembang City, South Sumatra Province, Indonesia”. This study has been conducted or will be conducted???
2. The authors stated the Palembang City was chosen as the study location because it has a large population of pregnant women and socio-cultural variations that reflect the diversity of urban community contexts in Indonesia. Please add the specific number or rank compared to other cities or provinces in Indonesia with reference
3. Is there any specific reasons for choosing those public health centers as your study site?
4. Please add the ethical clearance number and the institution issued the letter
5. The authors stated developed and validated the instrument in Oct 2024, which instrument which developed and validated? And what kind of validity and reliability test that you conducted?
6. “In the intervention phase, the authors involving the administration of music therapy and hypnobirthing sessions for four weeks under the supervision of **certified health personnel**”, who were they??
7. The authors used randomization for a quasi-experimental study design??
8. How did you know the respondents had psychiatric disorders so you excluded them?
9. The inclusion criteria were as follows: third-trimester pregnant women, experiencing moderate to severe lower back pain. Why you chose the pregnant women who had severe LBP? Did they take the medication to relieve the pain? It should be clear in the inclusion criteria because it could recall the bias of your outcome.
10. For the hypnobirthing intervention, please elaborate the intervention, how the technical steps of that intervention. When the respondents practice this intervention? At home? Alone? Assisted by whom? How you know the respondents did it correctly?
11. I did not see any explanation about the combined intervention part. It should be clearly stated with detail steps.

Results:

1. Don’t repeat this sentence “The research phase began with the approval of the ethics committee and research permit in July 2024. Researchers began to identify respondents based on the following inclusion criteria: pregnant women in the third trimester, experiencing mild to severe lower back pain, and primigravida. After the respondents were collected, lower back pain was measured using a visual analogue scale (VAS) questionnaire on 200 respondents of pregnant women in the third trimester in the working areas of the Pembina Health Center, Nagaswidak Health center, Taman Bacaan Health Center and Pembina Health Center”. It has been stated in the methods part
2. Please add the baseline data analysis for those 4 groups, is there any differences between groups?
3. P=0.000 should be written as p<0.001

Discussion

1. All interventions have a significant reduction in pain. However, the combined intervention had a higher reduction score compared to other groups. How you discuss it??

Is the work clearly and accurately presented and does it cite the current literature?

Yes

If applicable, is the statistical analysis and its interpretation appropriate?

Partly

Are all the source data underlying the results available to ensure full reproducibility?

Yes

Is the study design appropriate and is the work technically sound?

Yes

Are the conclusions drawn adequately supported by the results?

Yes

Are sufficient details of methods and analysis provided to allow replication by others?

Partly

Reviewer Expertise:

Health policy, public health nursing, healthcare management

I confirm that I have read this submission and believe that I have an appropriate level of expertise to confirm that it is of an acceptable scientific standard, however I have significant reservations, as outlined above.

# Response

**Published:** 2025-10-30

**Authors:** Murbiah murbiah

**Affiliations:** Nursing, Lincoln University College Faculty of Medicine, Petaling Jaya, Selangor, Malaysia

Dear Reviewer

 We would like to sincerely thank the reviewer for the thoughtful and constructive feedback provided on our manuscript entitled “Effectiveness of Hypnobirthing, Music Therapy, and Combined Intervention in Reducing Low Back Pain Among Third-Trimester Pregnant Women: A Quasi-Experimental Study.” Below we provide a detailed response to each point raised:

 1. Abstract

     a. We have added information about the location and time of the study to the Abstract and Methods sections, namely: *“*The study was conducted at four Community Health Centers (Puskesmas) in Palembang City, South Sumatra Province, Indonesia. **2. The statistical analyses used included the Paired Sample t-test, One-Way ANOVA, and Post hoc comparisons using Tukey HSD test for mean pain scores between the groups. This information has been added to the abstract section of the Methods section

    b. The conclusion section has been clarified to align with the research objectives. It now reads: “Music therapy and hypnobirthing have been proven effective in reducing lower back pain intensity in pregnant women in their third trimester, with the combination of both therapies providing the most significant effect.”

 2. Introduction 

 We have added an explanation in the Introduction section that this research is important to be conducted in primary health care facilities because most pregnant women in Indonesia receive antenatal care at community health centers. The implementation of non-pharmacological therapies such as music and hypnobirthing at the primary level can strengthen promotive-preventive services and reduce dependence on analgesics during pregnancy.

 3.Methods

    a. We have revised the sentence to the past tense: “This study was conducted in four primary health care centers (PUSKESMAS) spread across the administrative area of Palembang City, South Sumatra Province, Indonesia. Palembang

    b. We have added data from the South Sumatra Health Profile for the city of Palembang in 2024, which shows that Palembang has approximately 27,775 pregnant women, ranking first in South Sumatra Province. This information was included with official references from the Health Office.

    c. The four health centers were selected purposively because they had high numbers of antenatal visits, represented urban and suburban areas, and had health workers certified in hypnobirthing and music therapy. This explanation has been included in the Methods section.

    d. This study was approved by head of ethics review committee Dr. Suzanna, S.Kep., Ns., M. Kep on 25 November 2024 by Health Research Ethics Committee of IKesT Muhammadiyah Palembang have granted ethical approval No: 002009/KEP IKesT Muhammadiyah Palembang/2024).

    e. The instruments developed were the Music and Hypnobirthing Side Effect Checklist (MHSEC). Content validity was tested using the Content Validity Index (CVI = 0.92) with three experts, while reliability was tested using Cronbach’s Alpha (α = 0.84). This information has been clarified in the Methods section

    f. Interventions are carried out under the supervision of a certified hypnobirthing midwife who has received official training from an accredited institution

    g. The process of allocating participants into four groups was carried out using a computer-based random number generator prepared by an independent researcher. Participant identification numbers were entered into a list and randomized automatically using the Research Randomizer application (https://www.randomizer.org/). The randomization results were sealed in sequential sealed envelopes to maintain the confidentiality of the allocation

    h. Respondents with moderate to severe pain were selected because clinically this condition requires non-pharmacological intervention to avoid the use of analgesics. We confirmed that all respondents did not take analgesic medication during the study period, as stated in the exclusion criteria

   i. Hypnobirthing intervention is carried out in accordance with standard procedures included guided breathing, visualization, and deep relaxation techniques conducted by a certified practitioner Each hypnobirthing session lasts 30 minutes twice weekly for four week.

   j. Combination group: received a joint intervention combining hypnobirthing techniques and therapeutic music under the same schedule and procedures. The combined intervention included hypnobirthing sessions accompanied by relaxing instrumental music played at a slow tempo of 50–70 bpm under the supervision of certified health professionals.

 4.Result

 Duplicate sentences have been removed from the Results section to avoid redundancy. The statistical notation has been corrected throughout the manuscript to p < 0.001.

 5. Discussion

 The Discussion section has been expanded with an explanation that the synergistic effects of music therapy and hypnobirthing enhance the parasympathetic relaxation response and lower cortisol levels, thereby optimizing pain perception reduction

 We are deeply grateful for the reviewer’s constructive feedback, which has substantially improved the rigor and clarity of our manuscript. We have revised the paper accordingly and believe these changes address all concerns raised.

 Thank you for your valuable time and insightful review.

 warm regard

 murbiah and team

# Reviewer report: Reviewer response for version 1

**DOI:** 10.5256/f1000research.186071.r412884

**Published:** 2025-10-06

**Authors:** Abu R. Srinivasan

**Affiliations:** 1 Sri Balaji Vidyapeeth (Deemed to be University), Pillayarkuppam, Puducherry, India

Did the authors measure the adverse effects of music therapy at all??? If so, did they prepare self-report questionnaires and rating scales (duly validated) to assess psychological and  physiological data,prior to and following treatment??. The authors must please note that clinical observation and client interviews are also regarded crucial for documenting symptoms such as irritability, boredom, or even the exacerbation of the existing condition.

 Likewise, did the authors collect data through participant self-reports for assessing symptoms such as dizziness or anxiety and any other pertinent clinical observations.As an extension of the endeavour, then data analysis could be completed by computing the rate of adverse events, with particular reference to serious vs. non-serious effects as well as hypnosis-related vs. other possible causes.

 Outcome

 The primary outcome of the study was the reduction in lower back pain intensity before and after a four-week intervention period........Can this sentence be written without any ambiguity???

Is the work clearly and accurately presented and does it cite the current literature?

Partly

If applicable, is the statistical analysis and its interpretation appropriate?

Partly

Are all the source data underlying the results available to ensure full reproducibility?

Yes

Is the study design appropriate and is the work technically sound?

Partly

Are the conclusions drawn adequately supported by the results?

Partly

Are sufficient details of methods and analysis provided to allow replication by others?

Partly

Reviewer Expertise:

Biochemistry related to metabolism and metabolic disorders, Endocrine Biochemistry, Music as an adjuvant therapeutic modality, Diagnostic enzymology

I confirm that I have read this submission and believe that I have an appropriate level of expertise to confirm that it is of an acceptable scientific standard, however I have significant reservations, as outlined above.

# Response

**Published:** 2025-10-29

**Authors:** Murbiah murbiah

**Affiliations:** Nursing, Lincoln University College Faculty of Medicine, Petaling Jaya, Selangor, Malaysia

Dear Reviewer,

 We sincerely thank the Reviewer for this valuable input and for emphasizing the importance of monitoring potential side effects associated with the intervention. Below we provide a detailed response to each point raised:

 1. Measurement of Side Effects  

 Thank you for your valuable comments. In this study, the side effects of the intervention were observed using the Music and Hypnobirthing Side Effect Checklist (MHSEC). 

 Music and hypnobirthing Side Effect Checklist (MHSEC) developed and validated by researchers (I-CVI = 1.00; Cronbach's Alpha = 0.926). This instrument assesses physical and psychological symptoms that may arise during the intervention, including dizziness, nausea, headache, restlessness, palpitations, drowsiness, distraction, back pain, abnormal fetal movement, and mood. Observations were made before and after each therapy session by trained observers to ensure the safety of the intervention.

 We have added an explanation that adverse event data collection was conducted through participant self-reports and observations. Adverse event severity analysis has also been added to the results section, with classifications of “mild,” “moderate,” and “severe.” No severe adverse events were found in any of the intervention groups.

 2. Data interpretation

 Thank you for your suggestion.The sentence has been revised to be clearer and more specific about the main results and the intervention period. The main results of the study showed a significant reduction in lower back pain intensity after four weeks of intervention in all treatment groups, with the greatest reduction in the combination group of hypnobirthing and music therapy compared to the control group (p < 0.001).

 3. literature.

 Thank you for the reviewer's attention to the literature aspect. We have updated and expanded the bibliography by including the latest literature published between 2018 and 2023, covering experimental research results, meta-analyses, and systematic reviews relevant to the topics of hypnobirthing and music therapy.

 We are deeply grateful for the reviewer’s constructive feedback, which has substantially improved the rigor and clarity of our manuscript. We have revised the paper accordingly and believe these changes address all concerns raised.

 Thank you for your valuable time and insightful review.

 warm regard

 murbiah and team

# Reviewer report: Reviewer response for version 1

**DOI:** 10.5256/f1000research.186071.r412881

**Published:** 2025-09-16

**Authors:** Dinesh C. Sharma, Jyoti Sharma, Sumbul Zehra

**Affiliations:** 1 Bio musicology or Music Therapy, Km. Mayawati Government Girls P.G. College, Badalpur, Uttar Pradesh, India; 2 Research Associate (DST-CURIE) Dept of Bio musicology, Km. Mayawati Government Girls P.G. College, Badlapur, Uttar Pradesh, India; 3 Indian Association of Music Therapy, Delhi, Delhi, India

**Summary of the Article**

 The manuscript titled " *Effectiveness of Hypnobirthing, Music Therapy, and Combined Intervention in Reducing Low Back Pain Among Third-Trimester Pregnant Women: A Quasi-Experimental Study*" presents a quasi-experimental investigation involving 200 third-trimester pregnant women recruited from four primary health care centers in Palembang, Indonesia. Participants were randomly allocated into four groups: hypnobirthing, music therapy, combined therapy, and control (n=50 each). Pain intensity was assessed using the Visual Analog Scale (VAS) before and after a four-week intervention.

 The results indicate a statistically significant reduction in pain intensity across all intervention groups compared to the control (p < 0.001), with the combined intervention group showing the greatest improvement. The authors conclude that both hypnobirthing and music therapy are effective, and their combination provides synergistic benefits, supporting the use of these interventions as part of routine antenatal care.

**Strengths**

 •    The study addresses a significant clinical problem , pregnancy-related low back pain, which affects quality of life and labor outcomes.

 •    Use of a quasi-experimental design with four groups provides good comparative power.

 •    Incorporation of traditional Indonesian music is an important contribution to culturally contextualized maternal care research.

 •    The study reports proper ethical approval, informed consent, and anonymization of data.

**Points for Improvement**

 •    While the paper mentions "random group allocation," it does not describe how randomization was implemented (lottery method, random number generator, sealed envelopes?).

 •    Details on standardization of hypnobirthing sessions (duration, script used, practitioner training) and music therapy sessions (volume, environment control) are insufficient. Authors should specify steps taken to ensure intervention fidelity and reproducibility.

 •    The control group received "standard antenatal care with a leaflet." Please clarify whether attention control was ensured to reduce placebo effects (e.g., similar time spent with staff as intervention groups).

 •    Future research directions could include multi-center RCTs, cost-effectiveness studies, and assessment of psychological well-being outcomes.

**Overall Recommendation**

 The study addresses a relevant problem and presents promising findings with potential implications for maternal health practice. However, ** minor revisions** are required before the article can be considered scientifically sound and can be indexed.

**Decision:**

**Minor Revision Required**

 Summary of Required Revisions:

 •    Clarify randomization method, intervention fidelity, and control group comparability.

 •    Include confidence intervals and more detailed statistical reporting.

 •    Expand discussion of study limitations and suggest future research directions.

Is the work clearly and accurately presented and does it cite the current literature?

Yes

If applicable, is the statistical analysis and its interpretation appropriate?

I cannot comment. A qualified statistician is required.

Are all the source data underlying the results available to ensure full reproducibility?

Yes

Is the study design appropriate and is the work technically sound?

Yes

Are the conclusions drawn adequately supported by the results?

Yes

Are sufficient details of methods and analysis provided to allow replication by others?

Yes

Reviewer Expertise:

Bio-musicology, Music Therapy, Complimentary and Alternative Medicine, Breast Cancer

We confirm that we have read this submission and believe that we have an appropriate level of expertise to confirm that it is of an acceptable scientific standard.

# Response

**Published:** 2025-10-19

**Authors:** Murbiah murbiah

**Affiliations:** Nursing, Lincoln University College Faculty of Medicine, Petaling Jaya, Selangor, Malaysia

We would like to sincerely thank the reviewer for the thoughtful and constructive feedback provided on our manuscript entitled *“Effectiveness of Hypnobirthing, Music Therapy, and Combined Intervention in Reducing Low Back Pain Among Third-Trimester Pregnant Women: A Quasi-Experimental Study.”*

 We appreciate the reviewer’s positive assessment of our study’s clinical relevance, methodological design, and cultural contribution. Below we provide a detailed response to each point raised:

**1. Clarification of Randomization Method**

 Thank you for your very constructive feedback. We have clarified the randomization procedure in the Research Methods section. The allocation process was conducted using a computer-based random number generator via the Research Randomizer application. The randomization results were placed in sealed envelopes in sequential order to maintain the confidentiality of the allocation. (see the sample size section).

**2. Intervention Fidelity and Standardization**

 We agree with this comment. In the Intervention Procedures section, we have added the following information:  Hypnobirthing interventions are conducted according to standard procedures that include guided breathing techniques, visualization, and deep relaxation performed by certified practitioners. Each Hypnobirthing session lasts 30 minutes, twice a week for four weeks

 Music therapy sessions use music  Music therapy interventions are carried out in accordance with standard procedures, namely listening to soothing instrumental music for 30 minutes every day for four weeks, with a slow tempo of 50–70 beats per minute, moderate music volume, and a quiet environment. This aims to maintain the consistency of participants' sensory experiences. (Saad & Jariyah, 2022), the music used is the traditional composition “Gending Sriwijaya”. This work, created by Nungcik AR in 1943 and accompanied by Palembang-style gamelan music arranged by A. Dahlan Rachim and R.C. Hardjosubroto, is a symbol of South Sumatran culture. The traditional Gending Sriwijaya music used in this study is part of the traditional cultural heritage of the Palembang community in South Sumatra, Indonesia.

**3. Control Group and Attention Control**

 Thank you for your valuable suggestion. We have added a note that the leaflet was given once at the beginning of the intervention (week 1) and briefly discussed by the staff as a form of educational control to control group. The control group received 30-minute light education sessions conducted by health workers, so that the duration and attention received were comparable to the intervention group. The educational material consisted of information in the form of leaflets about hypnobirthing and music therapy. 

**4. Statistical Reporting and Confidence Intervals**

 We have revised the Results section by adding a 95% confidence interval (CI) and effect size to Table 5.

**5. Study Limitations and Future Directions**

 We have followed up on this input by adding a new paragraph in the Discussion section. We highlight the limitations of this study include the quasi-experimental design, which did not fully control for selection bias, and limitations in the subjective measurement of pain using the VAS. Further research is recommended in the form of a multi-center randomized controlled trial (RCT) and a cost-effectiveness analysis to assess the economic benefits of this intervention in routine antenatal care.

 We are deeply grateful for the reviewer’s constructive feedback, which has substantially improved the rigor and clarity of our manuscript. We have revised the paper accordingly and believe these changes address all concerns raised.

 Thank you for your valuable time and insightful review.

 warm regard

 murbiah and team
